# The Influence of Selected Solid Lubricants on the Wear of the Rolling–Sliding Interface in the Wheel–Rail System According to the Standard PN-EN 15427-2-1:2022

**DOI:** 10.3390/ma18071672

**Published:** 2025-04-05

**Authors:** Wioletta Cebulska, Henryk Bąkowski, Damian Hadryś

**Affiliations:** Faculty of Transport and Aviation Engineering, Silesian University of Technology, Krasińskiego 8, 40-019 Katowice, Poland; henryk.bakowski@polsl.pl (H.B.); damian.hadrys@polsl.pl (D.H.)

**Keywords:** green tribology, biodegradable materials, solid grease, soybean grease, graphite grease, molybdenum disulfide

## Abstract

This article presents the influence of lubricant on selected tribological properties of the rolling–sliding association, i.e., the wheel–rail system. Three solid lubricants were tested: soybean grease, molybdenum disulfide and graphite grease. Under specific operating conditions, a beneficial influence of lubrication of the above-mentioned friction node was observed. This is valuable information for rolling stock owners, track operation or maintenance workers when making decisions about lubrication or its absence on a given section of railway track. In this way, tangible financial benefits (savings) are obtained by extending the durability of the wheel rim and rail, and, through extended periods of wheel set reprofiling, we significantly reduce operating costs. Solid lubricants (lubricating sticks) intended for the lubrication of railway wheel flanges must meet the requirements specified in the PN-EN 15427-2-1:2022 standard. Annex H. The wear patterns were observed and analyzed using both optical microscopy and scanning electron microscopy (SEM) combined with energy-dispersive X-ray spectroscopy (EDS). The test results indicate that graphite is characterized by the lowest and most stable coefficient of friction over time, which makes it the most effective lubricant in terms of friction reduction. Soybean grease also shows stability and a low level of friction, but with a slight increase in value over a longer period of time. However, grease containing molybdenum disulfide, despite its initial effectiveness, loses its lubricating properties over time, resulting in a significant increase in friction.

## 1. Introduction

In real working conditions, stresses and slips occur in the wheel–rail contact area, which affect the processes occurring in this important place. The contact surface is subject to permanent changes depending on many factors, both design and operational. As a result of the overlap of these factors, the problem of the durability of the surface layer, especially the running surface of railway rails, remains unsolved [1,2]. In order to improve the conditions of cooperation between the wheel and the rail, especially when the rolling stock is running on small-radius curves (lateral slips), lubrication of the places most exposed to abrasive wear is used. The causes of this wear are tangential and normal stresses occurring at the contact point between the wheel and the rail. These stresses reach the highest values in traction (train) vehicles. For this reason, lubrication devices are installed in locomotives and railcars. The extreme sets of the vehicle (the first and the last) are always lubricated. Lubrication reduces the coefficient of friction and reduces the intensity of wheel wear [2] on the side surface of the rail head and the wheel flange.

The types of solid greases used to lubricate rail wheel flanges include biodegradable greases, such as soybean greases, which are environmentally friendly and effectively minimize wear, although they require more frequent application. Greases based on molybdenum disulfide (MoS_2_) are characterized by high resistance to loads and temperatures, creating a durable protective layer, although they can lose their properties over time. Graphite greases, thanks to their layered structure, effectively reduce friction and are resistant to high temperatures and water. Greases based on hexagonal boron nitride (h-BN) are distinguished by a low coefficient of friction, chemical stability and high resistance to degradation. The selection of the appropriate grease depends on the operating conditions, such as temperature, loads and environmental requirements.

**Figure 1:**
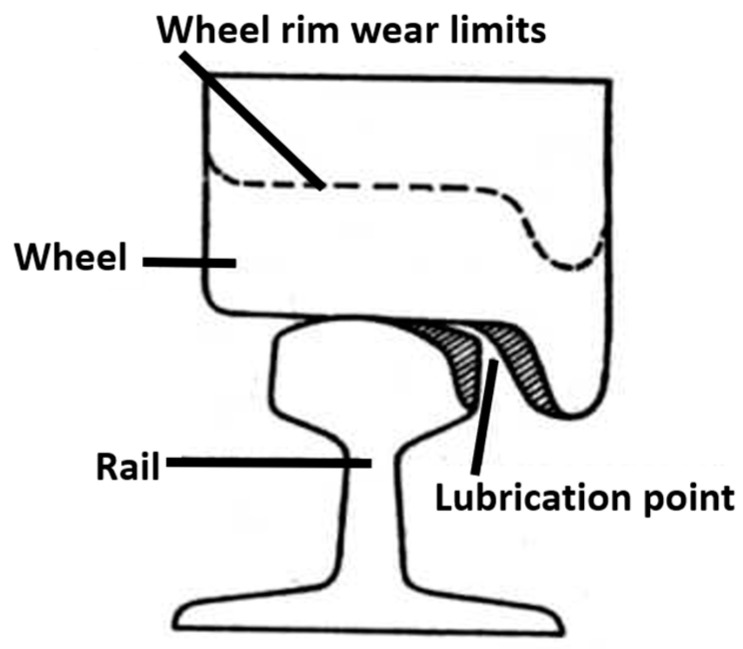
Areas of railway wheel lubrication and permissible wheel and rail wear [2,3].

Solid greases (lubricating sticks) intended for the lubrication of railway wheel flanges must meet the requirements of the PN-EN 15427-2-1:2022 Annex H standard [2,3]. It specifies the permissible value of the friction coefficient (≤0.15). Lubrication is used only at the lateral contact of the wheel flange with the inner surface of the rail head (Figure 1), and the method of supplying the lubricant prevents it from getting onto the running surface of the wheels and rails. This is important to not reduce the adhesion of the wheels and rails on the surfaces on which the traction force and the friction force are developed during braking (longitudinal slips) [4,5].

Depending on the railway line profile, the intensity of wheel flange wear in locomotives varies. Studies conducted by various railway authorities have shown that locomotives without lubrication devices serving lines with a difficult profile have mileage between wheel rim reprofilings from 8000 to 35,000 km [3,4]. After introducing lubrication devices in these locomotives, the mileage between wheel set reprofilings was extended by 2–3 times under the same operating conditions. This resulted in a three-fold longer wheel rim life. An additional benefit from lubrication is reduced rail wear. On lines with many small radius curves, their durability was found to be even twice as long [4,5,6]. Wheel flange lubrication devices in locomotives have a beneficial effect on the operation of rolling stock. First of all, they allow for significant savings in high-quality steel used for the production of wheel rims (even up to 80% [3,4]) and rails. They reduce the workload associated with maintaining wheel sets, as they require less frequent reprofiling. The downtime of traction vehicles in locomotive sheds and repair plants is also reduced, which results from the reduced need for rolling and replacing tyres [4,5,6].

In addition, these devices contribute to savings in driving energy ranging from 5% to 15% by reducing resistance to motion, which also translates into reduced electricity or fuel consumption. Their impact on environmental protection is also an important aspect, as they reduce noise levels and enable the use of biodegradable lubricants [4,7,8].

These devices are characterized by low dead weight, and their intelligent electronic control allows for adjustment to various needs. Their use reduces the costs of maintaining the railway surface, including rail replacement, which shortens the time of track closures. Finally, they also contribute to increasing the safety of rail traffic by reducing the risk of derailment [4,7,8,9,10,11].

It is important to remember the influence of lubricant on braking distance. The solid lubricant remains on the friction surface of the rail from the point of the applicator, but only for a short distance because it is mechanically removed by the following train sets. The braking distance may be longer. Solid grease reduces the coefficient of friction between the wheel and the rail, which can reduce braking efficiency. If grease is applied incorrectly (e.g., to the rail head instead of to the side), it can lead to reduced wheel adhesion, causing skidding. Over-lubrication can result in incorrect operation of anti-skid systems (e.g., ABS in trains), extending braking time and distance. There are also positive aspects to using solid grease. Under certain conditions, solid greases can help reduce sudden friction spikes (“stick-slip”), which can improve the stability of the braking process. This can be beneficial, especially on curves, where wheel–rail contact is more complicated, and lubrication can provide smoother and more predictable traction control [12,13].

## 2. Research Material

The sample used for the tests was R260 rail steel with a pearlitic structure (classic, most commonly used), and the properties are given in Table 1 and Table 2.

**Figure 2:**
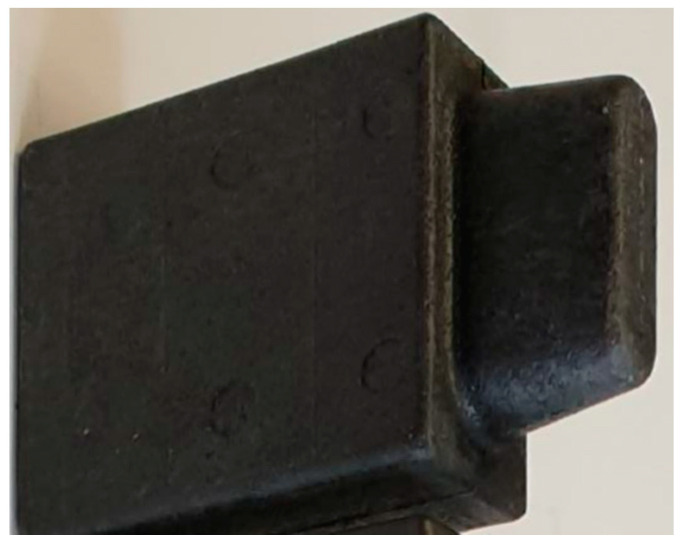
Example of a lubricating stick from Wojtowicz from Poland [14].

**Figure 3:**
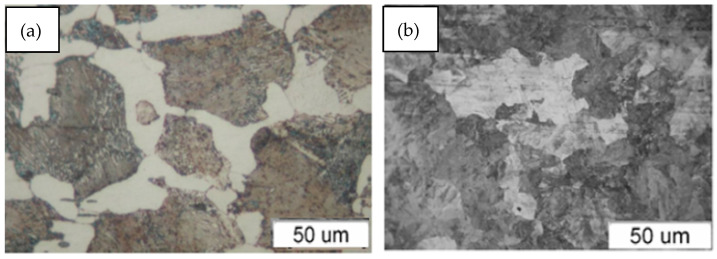
Microstructure of steel for wheel rims ((**a**)—visible pearlite grains with distinct ferrite phase boundaries) and railway rails ((**b**)—visible pearlite grains increasing wear resistance and plastic ferrite—lighter areas).

The sample cooperated with a counter-sample, with a shape made in accordance with the PN-PN-84/H-04332 standard [2,3]. In the rolling–sliding association, solid lubricant sticks with the addition of soy, molybdenum disulfide and graphite were used (Figure 2). In operating conditions, railway rails cooperate with steel for wheel rims (with a pearlitic microstructure with ferrite precipitations at grain boundaries—Figure 3). Counter-samples were made of the above-mentioned steel, and samples were made of R260 rail steel.

## 3. Research Conditions

**Figure 4:**
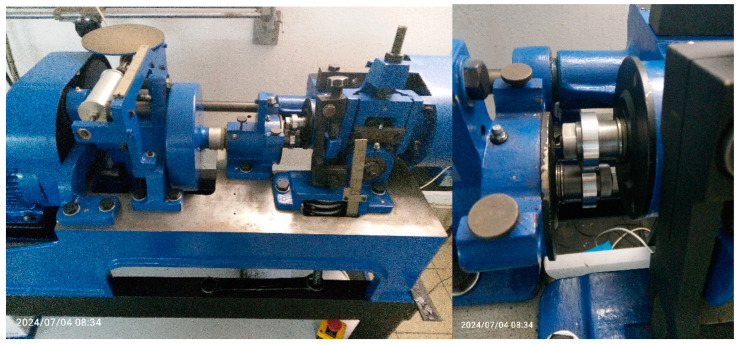
Testing device in a roll-to-roll configuration.

The test bench tests were carried out on a twin-disc machine tribological tester in a rolling–sliding contact under dry friction conditions. The roller–roller friction node, including the test bench, consists of a sample (rail steel with a pearlitic structure) and a counter-sample (bearing steel). The tests were carried out in accordance with the PN-EN 15427-2-1:2022 standard. The test bench is shown in Figure 4. As a result of the rotational movement of the rollers having a common contact area, the phenomenon of surface fatigue or abrasive/adhesive wear of the material can be observed (depending on the slip value). The conditions of cooperation were selected to reflect the actual pressures, slips and speeds in the wheel–rail contact on the selected track section in Silesia (pressure = 875 MPa velocity = 230 rpm, sliding = 10%). Tests were carried out on samples of rail steel with a pearlitic structure without heat treatment and with a hardness of 280 HB. The slip value adopted from the standard [2,3] was 10%. Rail steel samples were turned from the rail head and then polished to a surface roughness of Ra = 0.63 µm. The counter sample is made of rail wheel rim steel and has the same roughness as the sample. Comparing the compressive stresses generated in the contact zone during rolling and sliding friction of two cooperating elements, both in a real object and in a laboratory, it is possible to represent with a great approximation the conditions prevailing in both friction zones (Table 3). The slip value adopted from the standard [2,3] was 10%. During the tests at the twin on disc stand, the temperature was around 20 °C with a humidity of 60% [15].

**Figure 5:**
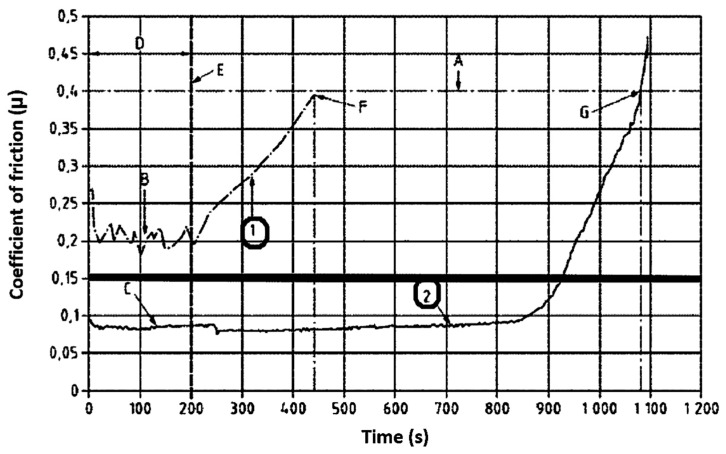
Dependence of the friction coefficient on the cooperation time recorded during tests according to the standard for two sample examples (1 and 2) [2].

Figure 5 shows a graph where curve 1 of the friction coefficient is used with a lubricant that does not meet the requirements of the assumed standard because it does not reach the limit value of the friction coefficient. Curve 2, on the other hand, is located below the value of 0.15 of the friction coefficient.

The tribological tests were conducted on the Amsler laboratory rig in accordance with the recommendations of the PN-EN 15427-2-1:2022 standard. Samples of the tested steel were made with a diameter of 38 mm, which worked with a counter-sample that was also in the form of a roller. Slip was obtained as a result of the appropriate selection of the geometric dimensions of the rollers. During the tests, the coefficient of friction was recorded. Table 4 presents the test parameters used during the experiment.

The graphical interpretation of the obtained results is shown in Figure 3. According to the PN-EN 15427-2-1:2022 standard, specific time zones were defined with the following characteristic points:

t_0_—moment of product application at μ ≥ 0.4;

t_1_—time at which μ ≤ 0.15 during the application of the solid lubricant sample according to the application process with a pressurized applicator;

t_2_—time of the product’s use (solid lubricant), i.e., the moment when the lubricant is removed (200 s);

t_3_—time when the friction coefficient reaches the value μ ≤ 0.15;

t_4_—time from the moment of reaching μ = 0.15 to μ = 0.4.

## 4. Research Results

**Figure 6:**
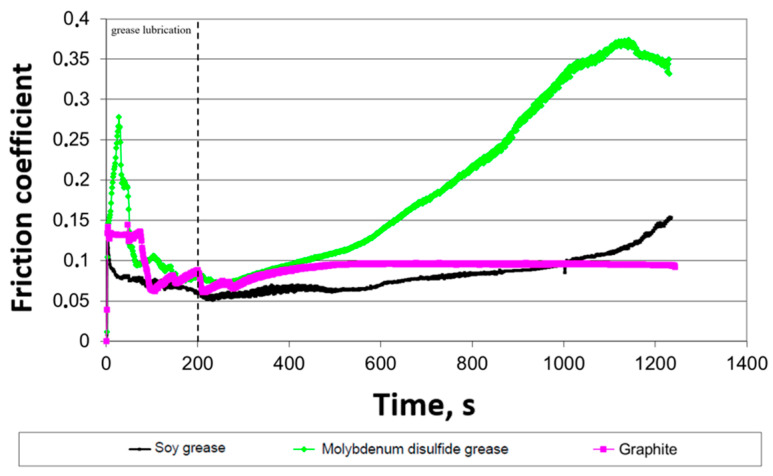
Coefficient of friction as a function of time in the rolling–sliding connection when lubricated with the tested plastic greases.

During the tribological tests, measurements were taken of two key parameters: wear, designated as (Δm), and friction torque (Mt). The obtained results enabled the analysis of the influence of friction conditions on the nature and intensity of wear of the tested elements. Figure 6 shows the friction coefficient of individual greases. The friction coefficient was determined based on a pair of forces realized on the device. On the one hand, a pendulum of length (*R*) was placed on the lower shaft, where the sample was placed, at the end of which a force transducer was placed. The friction force (T) appearing between the cooperating samples acts on the arm (*r*) with the length of the sample radius. Knowing the pressure (*F*) of the pendulum on the force transducer, we can determine the driving torque, which is balanced by the friction torque. The friction force is determined by the following formula:

(1) $$T=\frac{F*R}{r}$$

Then, the friction coefficient was determined as the quotient of the friction force (T) and the pressure force (*N*). The friction coefficient is determined by the following formula:

(2) $$µ=\frac{T}{N}$$

**Figure 7:**
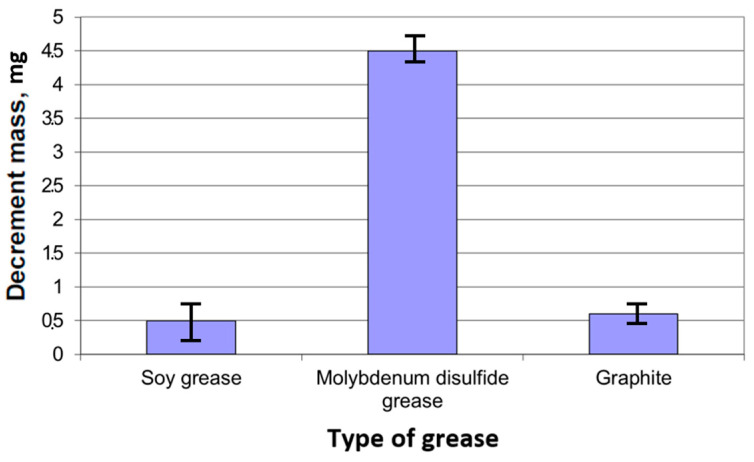
Weight loss of samples during friction in the presence of tested lubricants after 1200 s.

The tests were performed three times, and the average value is shown in Figure 6. This diagram shows the dependence of the friction coefficient on the interaction time for three different types of greases: soybean grease, molybdenum disulfide grease and graphite. The vertical axis represents the friction coefficient value, while the horizontal axis indicates time in seconds. The dashed line indicates the stick removed from the disc surface moment after 200 s. In the case of soybean grease, an initial stabilization of the friction coefficient at a level of about 0.1 is visible, followed by a slow increase over time. For molybdenum disulfide grease, a different course is observed: after an initial decrease, the friction coefficient begins to increase after 400 s and reaches a value exceeding 0.35 at the end of the measurement. Graphite, on the other hand, is distinguished by the lowest and most stable friction coefficient, which quickly stabilizes at a level of about 0.05–0.1 and remains almost unchanged throughout the experiment. Figure 7 shows the mass loss depending on the grease type.

Figure 7 shows the dependence of the sample mass loss on the type of grease used: soybean grease, grease containing disulfide and graphite grease. The bar chart contains confidence intervals that allow you to determine the range of values that contain the true value of a given parameter. The mass of the lubricant stick was measured before and after the tests, and the mass loss was calculated. Based on the tribological tests, it can be seen that the grease containing molybdenum disulfide shows the highest grease consumption mass loss, which was about 4.5 mg. In turn, soybean grease and graphite grease are characterized by much lower mass loss values. For soybean grease, the loss is about 0.5 mg, which is the lowest value of the three greases tested. Graphite grease shows a slightly higher loss, about 1 mg, but still much lower than the grease with molybdenum disulfide. In the case of supplying solid grease through an applicator, a lubricant film is formed on the mating surfaces. The test conditions (Table 3) for all greases were constant for all tested samples, which were specified in the PN-EN 15427-2-1:2022 standard Annex H.

## 5. Metallographic Research

### 5.1. Optical Microscopy

In order to determine precisely the changes occurring in the surface layer of the samples after tribological tests, a detailed analysis of the wear surface was carried out. The surface layer plays a key role in this type of research because it is on it and in its immediate surroundings that important physical and chemical processes occur, which differ from the properties of the core material. Observation of this layer provides valuable information on the mechanisms of wear and the reactions taking place, such as structural changes, oxidation or the formation of tribological products.

In order to determine the wear mechanism, metallographic tests were performed using an Olympus metallographic microscope, which allowed for detailed observation of the microstructure of the surface layer.

**Figure 8:**
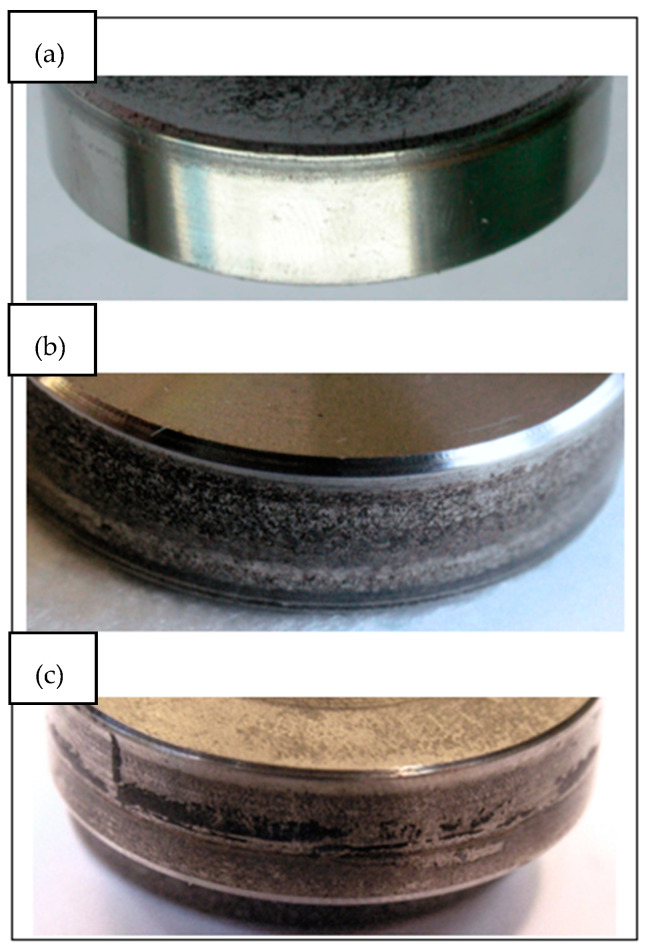
Macrophotographs (×8) of the roller surfaces after cooperation in the presence of soybean grease (**a**), MoS_2_ (**b**) and graphite (**c**).

In turn, macroscopic observations, due to their limited magnification, turned out to be insufficient for detailed analysis and interpretation of the results. They only allowed for a general assessment of the wear surface but did not allow for the identification of small details important for tribological analysis. Thanks to microscopic tests, precise information was obtained, which allowed for a better understanding of the processes occurring on the surface of the sample during tribological tests. Figure 8 shows a view of the roller surface.

**Figure 9:**
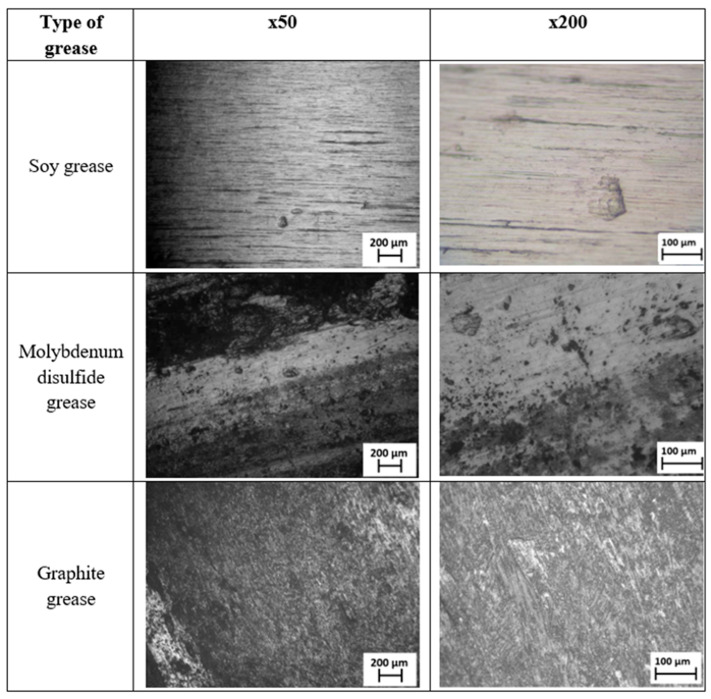
View of the roller surface after cooperation on the twin-disc machine stand in operating conditions according to the PN-EN 15427-2-1:2022 standard at ×50 and ×200 magnifications.

Figure 8 shows the surfaces of the samples after cooperation with solid lubricants on a macro-scale in order to assess their influence on the structure and nature of material wear. This analysis allows us to determine the effectiveness of lubricants in reducing friction and wear and to assess possible changes in surface topography after using different lubricants. Additionally, it allows us to assess the surface resistance to corrosion, which is crucial for determining the long-term durability of the material in various operating conditions. Figure 9 shows the surface of the tested rollers at magnifications of ×50 and ×200. There was a significant plastic deformation and number of holes on the surface of the sample lubricated with molybdenum disulfide compared to soybean and graphite grease.

Figure 9 shows surfaces lubricated with three different types of grease at two magnifications: ×50 and ×200.

The first grease tested, soy grease, left a relatively smooth surface with few defects. At higher magnification, small scratches and small deposits can be seen. This indicates a relatively mild effect of this grease on the material surface.

In turn, the grease with molybdenum disulfide caused more uneven wear, which is visible in the form of darker areas and numerous traces of use. There was clear damage on the surface, and the presence of molybdenum disulfide affected the structure of the protective coating.

The most uneven surface was left by graphite grease. The high roughness may be due to the specific properties of graphite, which, although it reduces friction, can also lead to characteristic changes in the material structure.

Analysis of these images shows that the soybean grease causes the least surface damage but may not provide as good protection as greases containing solid particles. The graphite grease is more temperature stable while maintaining a low coefficient of friction. It provides good surface protection against wear. High dustiness was observed during the use of this grease, which will be analyzed in further studies. The grease with the addition of molybdenum disulfide also appears to provide good wear protection, although it still leaves visible wear marks. The grease film is permanently removed during friction, so the wear of this grease is greater. The wear mechanisms of the samples differ because the graphite grease adheres better to the surface, creating a more effective lubricating layer, while molybdenum disulfide, despite its lubricating properties, adheres less well, leading to higher wear. Based on the differences in the chemical and physical structures of these materials, graphite has a layered structure that allows for easier adhesion to the surface while also allowing the layers to slide easily, which helps reduce friction and wear. Molybdenum disulfide, on the other hand, has a more compact structure, which means that it does not adhere as effectively to the surface, and its lubricating effect is less effective. Soybean grease, being an organic grease, works by creating a thin protective layer but may not provide as high friction resistance as mineral greases such as graphite or molybdenum disulfide due to its less stable structure at high temperatures. This type of analysis can be important in assessing the tribological properties of different greases and their effect on structural materials.

### 5.2. SEM EDS Research

Metallographic analysis of the friction surface of a rail steel sample was performed using a scanning electron microscope (SEM) to accurately assess the microstructure and type of wear of the surface layer. These studies allowed the identification of the material degradation mechanisms that occur during rail operation, such as adhesive wear, abrasion, and surface fatigue.

Before the analysis, the samples were carefully prepared—this included cutting, resin encapsulation, grinding, and polishing to obtain a perfectly smooth surface, enabling precise microscopic observation. Additionally, the surface of the sample was subjected to chemical etching to reveal the grain structure and possible material defects.

During the observation, photos were taken at different magnifications, which allowed for a detailed analysis of the surface topography and assessment of characteristic wear features, such as microcracks, abrasion marks, and the presence of oxide layers. The obtained images revealed surface heterogeneity, indicating local overheating, plastic deformation, and abrasion.

In addition to microscopic observations, chemical composition analysis was also carried out using energy-dispersive spectroscopy (EDS). This allowed us to determine the presence and distribution of chemical elements in the surface layer, which is crucial for understanding the tribological processes occurring at the wheel–rail interface. The analysis results showed the presence of surface contamination and traces of oxidation, which can contribute to accelerated wear.

**Figure 10:**
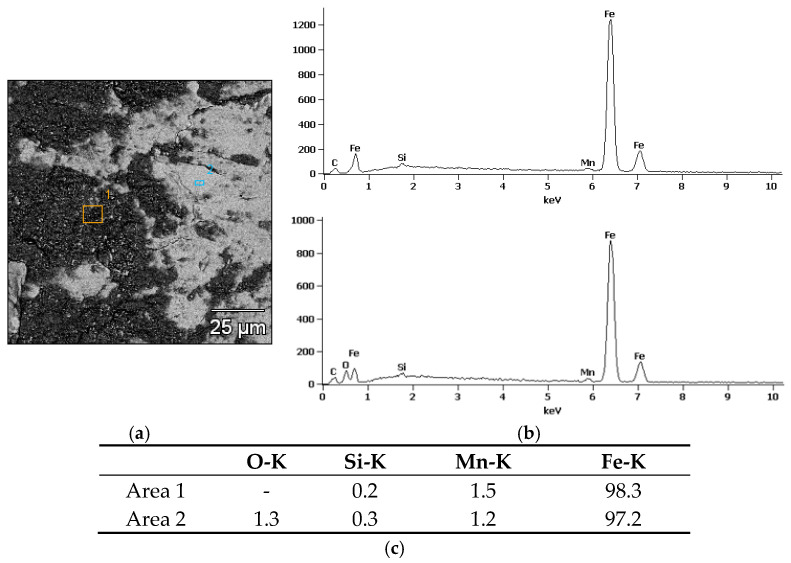
X-ray microanalysis of the chemical composition EDS of the friction surface after the roller made of rail steel with the use of soybean grease: (**a**) image with marked areas, (**b**) radiation spectrum (1–2), (**c**) table with quantitative analysis.

Analysis of the chemical composition of rail steel in the presence of various lubricants showed the presence of elements characteristic of the steel itself, such as iron (Fe), manganese (Mn), silicon (Si) and carbon (C). The first microanalysis (Figure 10), where soybean grease was used, showed elements for rail steel and oxygen (O). The presence of oxygen may indicate corrosion.

**Figure 11:**
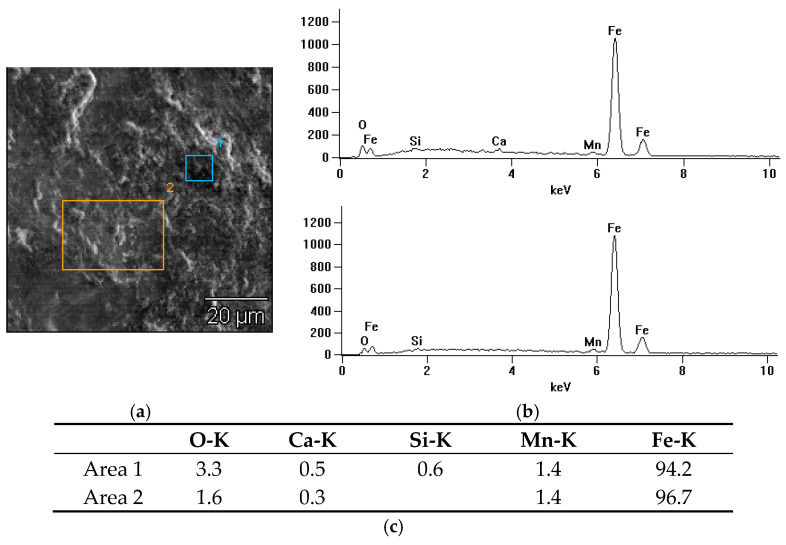
X-ray microanalysis of the chemical composition EDS of the friction surface after the roller made of rail steel with the use of molybdenum disulfide: (**a**) image with marked areas, (**b**) radiation spectrum (1–2), (**c**) table with quantitative analysis.

The second microanalysis spectrum (Figure 11), which shows the interaction of the rail with grease containing molybdenum disulfide (MoS_2_), shows the presence of elements that are part of rail steel and oxygen and calcium (Ca). The calcium content may be the result of the use of anti-wear additives or the origin of environmental pollution. A relatively high oxygen content may indicate an intensive oxidation process and degradation of the lubricant during operation.

**Figure 12:**
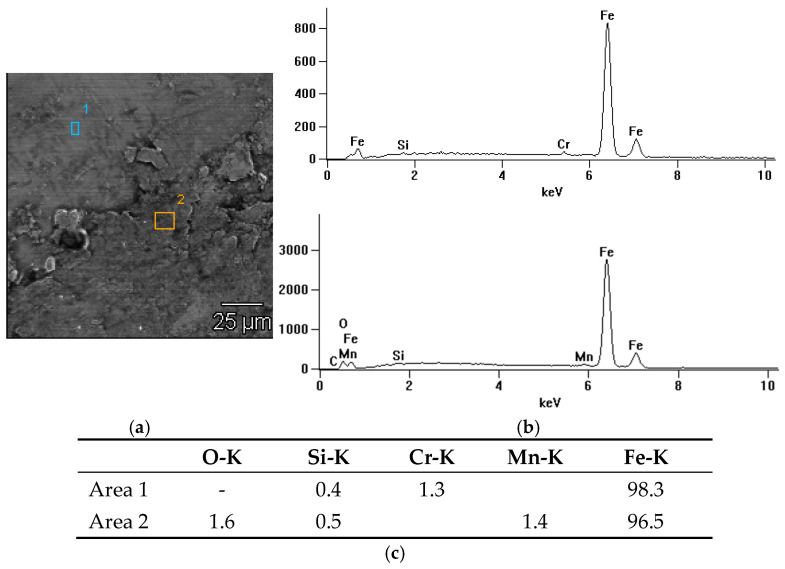
X-ray microanalysis of the chemical composition EDS of the friction surface after the roller made of rail steel with the use of graphite grease: (**a**) image with marked areas, (**b**) radiation spectrum (1–2), (**c**) table with quantitative analysis.

In the case of the third spectrum (Figure 12), which corresponds to graphite grease, the chemical composition includes elements that are part of rail steel and chromium (Cr), which may indicate additives that improve resistance to abrasion.

In summary, soy grease shows a moderate content of oxygen and carbon, which suggests some oxidation but also the presence of organic components. Unlike other greases, it does not contain chemical additives that improve lubricity, which makes it more ecological and environmentally friendly. MoS_2_ grease has a tendency to oxidize under operating conditions, which may affect its effectiveness over a longer period of use. Graphite grease, on the other hand, protects the surface well against oxidation, which makes it an effective protective agent in conditions with intense use.

## 6. Conclusions

Based on the conducted research and analyses, important conclusions were formulated regarding the use of lubricants in the wheel–rail system and the results of tribological tests. Lubrication of the rolling–sliding contact, especially in track curves, leads to a significant reduction in the wear of cooperating surface pairs and a reduction in the coefficient of friction. Thanks to the use of appropriate lubrication devices, it is possible to reduce the maintenance costs of the wheel–rail system by reducing the wear of wheel rims and rails and extending the periods between reprofiling. Tribological tests carried out on the Amsler stand in accordance with the PN-EN 15427-2-1:2022 standard showed clear differences in the behaviour of the tested lubricants. The test results indicate that graphite is characterized by the lowest and most stable coefficient of friction over time, which makes it the most effective lubricant in terms of friction reduction. Soybean grease also shows stability and a low level of friction, but with a slight increase in value over a longer period of time. However, grease containing molybdenum disulfide, despite its initial effectiveness, loses its lubricating properties over time, resulting in a significant increase in friction.

It is also worth nothing that the technological process of producing solid lubricants, especially those based on waxes, synthetic oils or modified resins, may result in the local accumulation of modifiers (such as thickeners, viscosity modifiers, anti-wear additives, antioxidants or substances improving lubricating properties). The accumulation of these modifiers in some areas of the material may result from several factors, such as improper miscibility of components, temperature gradients during the process or inhomogeneity during dissolution and the distribution of additives. This may lead to the uneven distribution of modifiers throughout the volume of the lubricant, which consequently affects its final properties, such as viscosity, durability or effectiveness in various operating conditions.

The analysis of mass loss confirms these observations. Molybdenum disulfide grease causes the greatest surface wear, with a mass loss of approximately 4.5 mg. In turn, soybean grease shows the smallest mass loss (approximately 0.5 mg), which indicates its high efficiency in minimizing wear. Graphite grease reaches an intermediate value (approximately 1 mg), maintaining good protective properties.

To sum up, both soybean and graphite grease meet the requirements of the PN-EN 15427-2-1:2022 standard, with graphite grease distinguished by greater durability, which is a significant advantage in conditions of intensive use of the wheel–rail system. The use of appropriate greases and lubrication devices significantly contributes to improving efficiency, reducing maintenance costs and protecting the environment. Soybean grease is more environmentally friendly due to the use of vegetable oils, as it biodegrades more easily and does not contain potentially harmful additives that can pollute soil or water. In contrast, graphite greases and those based on molybdenum disulfide, despite their excellent lubricating properties, can have a greater impact on the environment due to the longer decomposition time and origin of the ingredients. Therefore, biodegradable greases, such as soybean, are becoming increasingly popular in the industry as a more ecological alternative.

Future research should focus on the optimization of lubricant composition, testing under extreme conditions, the use of nanotechnology, ecology, durability, noise reduction, alternative technologies, influence on other components and the safety and influence of lubricants on braking distance.

## Figures and Tables

**Table 1:** Chemical composition of the tested materials.

Chemical Composition (% wt.) of the Tested Steel	Rail Steel R260	On the Wheel Rims *
C	0.72	0.52
Mn	1.11	0.8
Si	0.28	0.4
P	0.026	-
S	0.014	-
Cr + Mo + Ni	-	0.05
V	-	0.06

* PN-EN 13262 + A1:2009.

**Table 2:** Mechanical properties of tested materials.

Mechanical Properties of Tested Rail Steels	Rail Steel R260	On the Wheel Rims *
Rm, [MPa]	973	820 ÷ 940
Re, [MPa]	515	≥520
A5, [%]	12	≥14
KCU, [J/cm^2^]	28	20
HB	282	233

* b PN-EN 13262 + A1:2009.

**Table 3:** Summary of stress values depending on the type of system [10].

Load in Conditions		Stress in the Wheel–Rail Connection *	Roller-to-Roller Tension *
Real	Laboratory		
100 kN/wheel	2000 N	836 MPa	875 MPa

* Stress values calculated from Hertz’s formulas.

**Table 4:** Summary of stress values depending on the type of system.

List of Operating Factors According to the Standard Surface Pressure, MPa	Slippage, %	Speed, Min^−1^	Lubrication	Friction Coefficient Measurement
875	10	230	First 200 s	yes

## Data Availability

The original contributions presented in this study are included in the article. Further inquiries can be directed to the corresponding author.

## References

[1] ZEUS Współpraca kół Lokomotyw z Szyną w Łukach o Małych Promieniach. http://zeus.krb.com.pl/?wspolpraca-kola-lokomotyw-z-szyna-w-lukach-o-malych-promieniach,151.

[2] (2022). Aplikacje Kolejowe—Smarowanie kół i Szyn—Wymagania Eksploatacyjne—Część 2-1: Urządzenia Stacjonarne.

[3] Wójtowicz A., Bąkowski H. (2013). Oszczędności wynikające ze smarowania obrzeży kół w łukach o różnym promieniu. Tech. Transp. Szyn..

[4] Srivastava J., Sarkar P., Meesala R.K., Ranjan V. (2018). A numerical study on effects of friction-induced thermal load for rail under varied wheel slip conditions. Simulation.

[5] Andersson R., Torstensson P.T., Kabo E., Larsson F. (2015). The influence of rail surface irregularities on contact forces and local stresses. Veh. Syst. Dyn..

[6] Deters L., Proksch M. (2005). Friction and wear testing of rail and wheel material. Wear.

[7] Szymański M. (2022). Szyny o mikrostrukturze bainitycznej. Probl. Kolejnictwa Railw. Rep..

[8] Descartes S., Saulot A., Godeau C., Berthier Y. (2011). Wheel flange/rail gauge corner contact lubrication: Tribological investigations. Wear.

[9] Zhang W., Yamashita S., Kita H. (2020). Progress in tribological research of SiC ceramics in unlubricated sliding—A review. Mater. Des..

[10] Vásquez-Chacón I.A., Gallardo-Hernández E.A., Moreno-Ríos M., Vite-Torres M. (2021). Influence of surface roughness and contact temperature on the performance of a railway lubricant grease. Mater. Lett..

[11] Lewis S.R., Lewis R., Evans G., Buckley-Johnstone L.E. (2014). Assessment of railway curve lubricant performance using a twin-disc tester. Wear.

[12] Rey T., Papin E., Fridrici V., Dassenoy F. (2025). Experimental study of the tribological performance of a solid lubricant stick with MoS_2_ for wheel-rail contact. Tribol. Int..

[13] Czyczula W. (2009). Report on the Rail Lubricurve 50 Rail Lubrication System (Appendix No. 1).

[14] AW Solutions Sztyfty Smarne. https://aw-solutions.pl/produkty/sztyfty-smarne/.

[15] Bąkowski H. (2014). Evaluation of wear processes in the rolling-sliding contact by means of flake wear debris. Monograph.

